# Protecting energy intakes against income shocks^☆^

**DOI:** 10.1016/j.jebo.2017.06.007

**Published:** 2017-09

**Authors:** Stephanie von Hinke, George Leckie

**Affiliations:** a University of Bristol, Department of Economics, UK; b University of Bristol, Centre for Multilevel Modelling, UK

**Keywords:** Nutritional intakes, Food expenditures, Income shocks

## Abstract

Whether and how changes in economic circumstances or household income affect individuals’ diet and nutritional intakes is of substantial interest for policy purposes. This paper exploits a period of substantial income volatility in Russia to examine the *extent to which*, as well as *how* individuals protect their energy intakes in the face of unanticipated shocks to household income. Using rich data from the Russia Longitudinal Monitoring Survey, our results suggest that households use substitution, disproportionally cutting back spending on non-foods to protect spending on foods, change the composition of the consumption basket, and increase the consumption of ‘cheaper’ calories. Taken together, however, we find that total energy intakes as well as the nutritional composition of the diet are almost fully protected against income shocks. Specifically, we find that 12–16% of the effect of permanent income shocks on food expenditures is transmitted to energy intakes, with 84–88% protected through insurance mechanisms.

## Highlights

• We study *whether* and *how* individuals protect energy intakes against income shocks.
• We find that households use substitution, both between and within spending categories.
• Total nutritional intakes are almost fully protected against income shocks.
• 12-16% of permanent income shocks on food spending is transmitted to energy intake.

## 1 Introduction

Changes in economic circumstances affect many individual and household decisions. For example, sudden shocks to income affect decisions with respect to consumption (Blundell et al., 2008), health behaviours (Adda et al., 2009), and investments in children (Carneiro and Ginja, 2012). We are interested in whether changes to the economic environment, and shocks to household income in particular, affect individuals’ energy intakes. There is much interest in this relationship; its understanding is essential in evaluating how certain policies, economic circumstances or shocks impact on household resources and affect individuals’ nutritional outcomes (see e.g. Ruhm, 2000). In addition, it is crucial in informing the design of social insurance and income maintenance schemes (e.g. tax reforms, cash transfers). Our starting point is that individuals have a steady-state daily energy intake, which they aim to keep constant.^1^ Finding a drop in energy intake in response to a fall in income therefore suggests that individuals do not have the resources to sustain their current energy intakes. Hence, in addition to examining *whether* income shocks affect energy intake, we investigate *the extent to which*, as well as *how* individuals smooth, or ‘insure’, their energy intake in the face of unanticipated shocks to household income. We use the term ‘insurance’ to denote any changes in behaviour aimed to protect, or keep constant, individuals’ energy intakes.^2^

Broadly speaking, there are three ways to insure energy intake in response to an income shock. First, as discussed in the consumption insurance literature (see e.g. Besley, 1995, Townsend, 1995, Heathcote et al., 2009, Attanasio and Weber, 2010), individuals can make adjustments to their savings and labour supply to ensure a constant energy intake. In the context of our study, however, these more ‘standard’ insurance mechanisms do not play a large role. Indeed, we exploit a period of substantial income volatility in Russia, which only saw small fluctuations in employment rates and hours of work, and where most households do not have financial assets or access to financial institutions such as banks or credit unions. In addition, any insurance against income shocks depends to a large extent on the structure of the welfare state and the country’s safety net, which was largely absent in Russia at the time (Curtis, 1996). Instead, we therefore focus on the other two mechanisms to insure energy intakes. With that, we add to a growing literature on how households adjust their food basket during recessions. First, individuals may use substitution, substituting non-food spending with food spending, as well as changing the composition of the food basket, replacing ‘more expensive’ calories with cheaper ones. This is closely tied to the food Engel Curve literature. Hence, although this concerns *substitution*, we use the term *insurance*, as the substitution reflects changes in behaviour that aim to protect energy intakes. Second, individuals may rely more on home produced foods, and on informal networks, such as family and friends (see e.g. Rosenzweig, 1988, Udry, 1994).

Our main contribution, therefore, is to examine the importance of these mechanisms. The absence of a labour supply response in this setting allows us to focus on the other (joint) mechanisms. We model both the household-level consumption response and individual-level nutrition response to income shocks. We not only explore differences in the consumption response of food versus non-food, but also differentiate between different food groups within total food spending. We do this within the partial insurance framework developed by Blundell et al. (2008), allowing for differential effects of permanent and transitory income shocks. To examine the individual-level nutrition response, we extend the partial insurance model, and investigate the effect of *household-level* income shocks on *individual-level* nutritional intakes, whilst (i) allowing for differential effects for different household members, (ii) allowing for clustering of individuals’ diets within households, and (iii) investigating the importance of positive versus negative income shocks. This allows us to quantify the proportion of the response in food expenditures that is transmitted to energy intakes. With that, we are able to evaluate the importance of alternative insurance mechanisms available to individuals.

The results show that households are able to smooth their energy intakes substantially. We find that 12–16% of the effect of permanent income shocks on food expenditures is transmitted to changes in energy intakes, with 84–88% insured through the various insurance mechanisms available to individuals. We find no significant difference in the response to permanent shocks for men compared to that for women, though there is some suggestion that men respond more to transitory shocks than women. It is important to note that we explore these issues in the context of a mostly overweight or obese society. Indeed, neither child nor adult undernutrition seems to be a problem in the Russian Federation, with overweight and obesity dominating all income quintiles (FAO, 2003).

Key to our analysis is the rich data we use, the Russia Longitudinal Monitoring Survey (RLMS), as well as its unique context. Indeed, the analysis to address our research questions requires rich large-scale longitudinal data, linking individual-level nutritional intakes over time to detailed information on their incomes and expenditures. Few such datasets exist: longitudinal datasets tend to either include detailed information on income with limited information on nutrition, or detailed information on nutrition with limited information on income. In addition, where longitudinal datasets do include nutritional intakes, they tend to report *household-level* energy intakes, whereas the relevant unit of analysis is the individual.^3^ The RLMS is unique in that it collects longitudinal data on *individual-level* energy and nutritional intakes, linked to data on income, as well as expenditures. Another advantage of these data is that they allow us to study a period of substantial income volatility. The radical, market-oriented reforms introduced in 1992 led to the collapse of the economy in the 1990s, with a recovery thereafter, leading to considerable variation in our variables of interest.

Our paper is closely linked to the food Engel curve literature, well summarized by Chai and Moneta (2010). Engel’s law states that the poorer the family is, the larger the budget share it spends on food. He argued that, when a family cannot satisfy all of its wants, it tends to sacrifice the higher-order wants such as clothing to satisfy more basic ones such as food (Engel, 1857). Our results are consistent with Engel’s law.

Our paper is also closely tied to the consumption insurance literature. Jappelli and Pistaferri (2010) discuss some of the main studies, and describe the different approaches used to estimate the consumption response to income shocks. We build on the framework developed by Blundell et al. (2008), which allows us to estimate the *degree* to which income shocks are transmitted to consumption, distinguishing between permanent and transitory shocks. We apply these methods to a different literature and research question, examining the extent to which energy intake is insured.^4^

Our paper is also closely linked to the literature that examines the responsiveness of nutrient intakes to changes in income. The majority of this literature focuses on developing countries, but Stillman and Thomas (2008) examine this using the RLMS.^5^ They find that energy intake is very resilient to ‘short-term’, but less to ‘longer-run’, variations in household resources. Hence, our results are generally consistent with their analyses.

Finally, our focus on the relationship between income and nutrition reflects a more recent interest in how the business cycle affects individuals’ food consumption and health more generally. For example, Ruhm, 2000, Ruhm, 2003, Ruhm, 2005 finds that individual health and health behaviours deteriorate in good economic times, though he finds no effect on the consumption of fruit and vegetables. Investigating the effects of the Great Recession, Griffith et al. (2015) find that UK households adjusted to the economic environment by switching to cheaper calories, increasing their shopping effort (proxied by the use of sales, the number of shopping trips) without lowering the nutritional quality of their groceries.

The paper is organised as follows. Section 2 provides a brief description of the volatility in Russia during the 1990s and early 2000, and Section 3 discusses the conceptual and empirical framework. We present the data and descriptive statistics in Section 4, followed by the results in Section 5. Section 6 reports the robustness analyses. Section 7 places our results in the wider literature, and Section 8 concludes.

## 2 Background

The Russian reforms in the 1990s were responsible for dramatic changes in the Russian economy, affecting many aspects of family life. We can roughly distinguish two time periods: the downturn pre-1998 and the recovery thereafter. The downturn is characterised by rapid price increases: the cost of the minimum subsistence basket surged from 1900 Rubles in 1992 to 411,200 Rubles in 1997 (O’Brien and Patsiorkovsky, 2006). Employers’ response to the downturn included two forms of hours adjustment, short-time and involuntary leave. Short-time implied reduced working hours and was usually of a temporary nature. In 1994, about 6% of employees were on short-time and 8% on involuntary leave, though many individuals had secondary jobs (World Bank, 1995). In fact, there was only a small drop in male and female employment rates, and hours of work remained stable. Even in the downturn, the average employee worked more than 40 h per week (Gorodnichenko et al., 2010). This suggests that the labour supply response to the economic reforms was limited, with little change in terms of employment rates and hours of work (see also Semenova and Thompson, 2004). Nevertheless, there were large decreases in incomes due to the vast reductions in real hourly wages of 10% per year, and wage payments were delayed three to five months on average.

In the recovery phase (approximately 1998 onwards), inflation stayed low by Russian standards at around 10–20% and earnings increased substantially: real hourly wages rose by 9% per year, again with little to no changes in hours of work, and with a relatively constant employment rate. In addition to the extensive income variation over time, there is considerable variation across regions. Involuntary leave in 1994, for example, ranged between 1% in some regions to 16% in others, and short-time varied between 0.1% and 13.5% (World Bank, 1995). Disparities in Russia are also far greater than those across states in the US: per capita income in the richest region in 2005 was 10.6 times higher than that in the poorest region. The comparable ratio in the US was 1.8 (Gorodnichenko et al., 2010).

The period of the reforms is also characterised by a general absence of a welfare state or safety net, with large groups of the population having no access to any benefits, leading to a growing proportion of Russia’s population living on the poverty threshold (Curtis, 1996). In addition, the quality of medical care provision was very low by Western standards, with epidemic diseases such as cholera and typhoid fever increasing significantly, and rates of tuberculosis, cancer and heart disease higher than any industrialised nation. Russia’s mortality rate reached its peak in the mid 1990s, with the drop in life expectancy attributed to the demise in the anti-alcohol campaign (Bhattacharya et al., 2013), rather than due to nutritional problems.

As ‘standard’ insurance mechanisms such as savings and labour supply did not play a large role in the period of the reforms, Russians had to protect their energy intakes in other ways. Indeed, the main source of insurance for Russians was through substitution, informal networks, and home production. For example, our data show that, in the mid-1990s, 67% of respondents indicate they cut down on buying clothes/shoes to adjust to the new living conditions, and 55% cut down on meals, suggesting that non-food to food substitution may be important. Similarly, informal exchange networks in Russia are an important source of goods and services: 76% of households in our sample indicate to either have given money or goods to or received these from friends, family members, strangers or organisations in the last 30 days. Finally, Struyk and Angelici (1996) estimate that approximately 25% of Russian families living in large cities in 1995 had a dacha (a small home outside the city partially intended for growing foods). Growing one’s own food is common in Russia, among both poor and affluent families, with 64% of our sample indicating to be engaged in some home production. This suggests that these alternative insurance mechanisms are important. We estimate the extent to which such mechanisms protect individuals’ energy intakes against unexpected shocks to income.

## 3 The conceptual and empirical framework

### 3.1 The conceptual framework

We consider the standard constrained household utility maximization model of consumption. If households can borrow and lend at a common interest rate, and if the utility function is state and time separable, we obtain the Euler equation for consumption:

$$u^{\prime }\left(c_{h,t}\right)={\left(1+\delta \right)}^{-1}E_{t}\left\{\left(1+r_{t+1}\right)u\text{'}\left(c_{h,t+1}\right)\right\}\text{,}$$

where $u^{\prime }\left(c_{h,t}\right)$ is the marginal utility of consumption for household *h* at time *t*, *δ* is the intertemporal discount rate, *E_t_* is the expectation conditional on all information available at time *t*, and *r* is the interest rate.^6^ At any time *t*, the household chooses its consumption conditional on all information available at that time. Assuming a quadratic utility function and *r = δ* gives the following martingale process:

$$E_{t}u^{\prime }\left(c_{h,t+1}\right)=u\text{'}\left(c_{h,t}\right)\text{.}$$

This implies that, ex ante, current marginal utility is the best predictor of the next period’s marginal utility, and ex post, marginal utility changes only if expectations are not realised (Hall, 1978, Jappelli and Pistaferri, 2010). We can write this as

(1) $$c_{h,t+1}=c_{h,t}+e_{h,t+1}$$

where *e_h,t+1_* is the innovation term that summarizes all new information available at time *t + 1*.

We assume that household income, *y*_*ht*_, is the main source of uncertainty. As implied by (1), *anticipated* changes in income do not affect the marginal utility of consumption, because the consumer incorporates the expected income change in the optimal consumption plan. In contrast, the marginal utility of consumption does change in response to *unanticipated* income shocks, where the extent of the change depends on the nature and duration of the shock, as well as the availability of any insurance mechanisms. As in Jappelli and Pistaferri (2010), we can rewrite (1) to examine changes in consumption as a function of the change in the expectation of future income:

$$\Delta c_{h,t+1}=\frac{r}{\left(1+r\right)}{\left\{1-\frac{1}{{\left(1+r\right)}^{T-t}}\right\}}^{-1}\overset{T-t}{\underset{\tau =0}{\sum }}{\left(1+r\right)}^{-\tau +1}\left(E_{t+1}-E_{t}\right)y_{h,t+\tau +1}\text{.}$$

Here the change in consumption between *t* and *t + 1* depends on revisions in the expectation of lifetime future income: $\overset{T-t}{\underset{\tau =0}{\sum }}{\left(1+r\right)}^{-\tau +1}\left(E_{t+1}-E_{t}\right)y_{h,t+\tau +1}$. If income is very persistent over time and in the absence of any insurance possibilities, all changes in income are permanent and the marginal propensity to consume from income shocks equals 1. In addition, the framework predicts that the response to permanent shocks is no different whether this is a positive or negative shock. Alternatively, if income is serially uncorrelated, there is no change in the household’s expectation of its future income stream, and consumption is much less volatile than income. The response to such transitory shocks, however, may be asymmetric depending on the constraints faced by households. That is, if households are credit constrained (i.e. they can save, but not borrow), they will cut consumption when hit by a negative transitory shock, but will not react to a positive one (Jappelli and Pistaferri, 2010). In reality, income will consist of both: a component that is persistent, as well as a component that is transitory; we model this below.

### 3.2 The income process

We model income as a stochastic process. To distinguish between the permanent and transitory components, we use the statistical framework introduced by MaCurdy (1982), and Meghir and Pistaferri (2004). We model log real disposable income as:

(2) $$y_{h,t}=Z{\text{'}}_{h,t}\beta_{t}+u_{h,t}^{Y},$$

where *Z_h,t_* denotes a vector of covariates, with the associated vector of year-specific *β*_*t*_ coefficients. We follow the existing literature (e.g. Blundell et al., 2008, Gorodnichenko et al., 2010, Blundell and Etheridge, 2010) and define the covariates as indicators for the number of household members, location characteristics (including an urban dummy, and indicators for Moscow and St. Petersburg, and the federal districts), a set of indicators for educational attainment of the adult household members, and a quartic polynomial in the age of the adult household members. Thus, $u_{h,t}^{Y}$ is log real disposable household income net of predictable components, including any macroeconomic (regional) variation. This therefore also accounts for any potential regional differences in (food) prices. We examine price trends and discuss whether they may affect our estimation in more detail below. We decompose $u_{h,t}^{Y}$ into the sum of a permanent, *P_h,t_*, and transitory, *v_h,t_*, component:

$$u_{h,t}^{Y}=P_{h,t}+v_{h,t}.$$

We follow the literature (see for example Blundell et al., 2008, Gorodnichenko et al., 2010) and assume that permanent income follows a martingale process:

*P_h,t_* = *P_h,t−1_* +* η_h,t_*

where ηh,t are permanent income shocks, assumed independently and identically distributed (iid) across h and t. Examples of such a shock are a promotion or some technological shock that makes one’s skills more or less valuable in the labour market, affecting not only contemporaneous income, but also that in the future. The transitory component is given by *v_h,t_*, which follows an MA(q) process:

$$v_{h,t}=\overset{q}{\underset{j=0}{\Sigma }}\theta_{j}\varepsilon_{h,t-j},\;\;\;\;\;\;\;\;\theta_{0}=1$$

where *θ_j_* denote the lag coefficients, *ε*_*h,t*_ denotes the iid transitory shocks, and the order *q* is set by examining the unexplained income autocovariances. Examples of transitory shocks can be involuntary leave, wage delays, a bonus, or a short illness that affects productivity on the job. We assume that the permanent and transitory income shocks have mean zero and are uncorrelated: $E\left(\eta_{h,t}\right)=E\left(\varepsilon_{h,t}\right)=E\left(\eta_{h,t}\varepsilon_{h,t}\right)=0$, for all *h = 1, …, H* and *t = 1994, …, 2005*. It follows that unexplained income growth $\left(\Delta u_{h,t}^{Y}=u_{h,t}^{Y}-u_{h,t-1}^{Y}\right)$ can be written as:

(3) $$\Delta u_{h,t}^{Y}=\eta_{h,t}+\Delta \varepsilon_{h,t}\text{.}$$

**Fig. B1:**
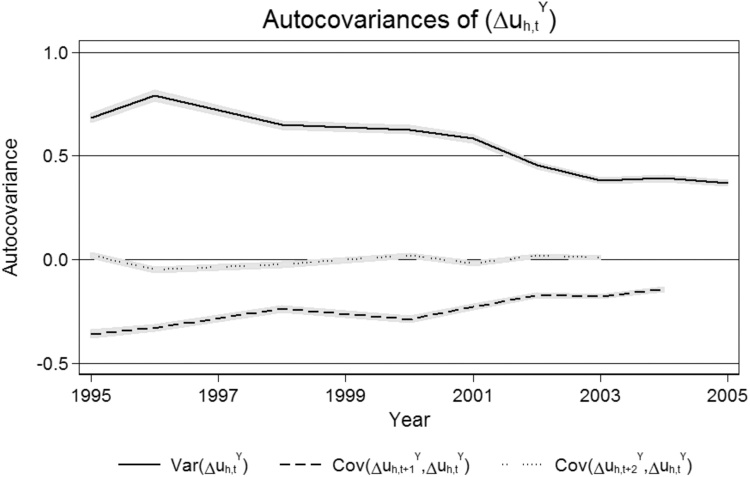
The autocovariance matrix of unexplained income growth.

The autocovariances of unexplained income growth, presented in Fig. B1 of Appendix B, suggest that an i.i.d. process fits the data well. Hence, in our discussion of the empirical methodology below, we set *v_h,t_* *= ε_h,t_*, as in Blundell et al. (2008), Gorodnichenko et al. (2010), and Carneiro and Ginja (2012), among others.

### 3.3 Income and expenditures

The conceptual framework, predicting a one-to-one response to permanent shocks, reflects a situation with no insurance possibilities. However, we argue that individuals have access to various mechanisms to insure their consumption, and that the response to income shocks is likely to differ for different *types* of consumption. Following Engel’s prediction, we therefore estimate the *degree* of transmission of income shocks to consumption of the following expenditure categories: foods, clothes/shoes, and other goods (the latter including durables, services, utilities, and fuel) whilst distinguishing between permanent and transitory shocks. For this, we follow the framework introduced by Blundell et al. (2008) and model the residual (unexplained) expenditure growth $\Delta u_{h,t}^{E}$ (obtained using the same approach as above and where the superscript *E* denotes expenditures) as a function of the income shocks. The model is written as:

(4) $$\Delta u_{h,t}^{E}=\phi^{E}\eta_{h,t}+\psi^{E}\varepsilon_{h,t}+\Delta \xi_{h,t}^{E}\text{,}$$

where the factor loadings *ϕ^E^* and *ψ*^*E*^ measure the responsiveness of expenditure growth to permanent and transitory income shocks, respectively. An estimate of one suggests that changes in income are fully transmitted to changes in consumption; the closer to zero the estimate, the better the insurance. The term $\Delta \xi_{h,t}^{E}$ denotes innovations in expenditure growth, which may capture measurement error, preference shocks, etc.

Our empirical approach can be summarized in two steps. First, we calculate the residual (unexplained) growths in income and expenditure ($\Delta u_{h,t}^{Y}$ and $\Delta u_{h,t}^{E}$); the annual changes in income and expenditure respectively, net of observable characteristics. Second, we estimate a system of year-specific household income and expenditure regression models with correlated errors, measuring and capturing the influence of permanent and transitory income shocks on the different expenditure categories. The key parameters are the variances of the permanent ($\sigma_{\eta ,t}^{2}$) and transitory ($\sigma_{\varepsilon ,t}^{2}$) income shocks, the variances of the innovations of the three expenditure categories ($\sigma_{\xi ,t}^{E}$), their covariances, and the insurance parameters *ϕ^E^* and *ψ*^*E*^ for each of the three expenditure categories. Appendix A discusses the identification in full.

We then investigate the composition of the food basket, i.e. whether the response to income shocks differs between different categories *within* total food spending. For this, we simultaneously model expenditures on the following food groups: grains, meat, dairy, fruit and vegetables, sweets, and beverages.

Note that we do not model potential changes in labour supply in response to income shocks as the period only saw small changes in both male and female employment rates, and in their hours of work (see Section 2). This suggests that the labour supply response to the economic reforms was limited (see also Gorodnichenko et al., 2010). We therefore treat this as exogenous, as in e.g. Krueger and Perri (2006) and Blundell et al. (2008).^7^

### 3.4 Income and the price of calories

Even if food expenditures reduce in response to a drop in income, energy intake may be unaffected if individuals have access to other mechanisms to sufficiently insure their energy intake. We start by exploring whether the *price* of calories responds to income shocks, proxied by the number of calories (kcal) consumed per Ruble spent on food (*energy_ih,t_*/*food expenditures_h,t_*), where *i, h*, and *t* denote the individual, household and year respectively.^8^ For this, we use the residual (unexplained) price per calorie, denoted by $\Delta u_{ih,t}^{PC}$, where the superscript *PC* denotes the price per calorie. We define *i = 1, 2* as the man and woman respectively.

Note that income shocks are measured at the household level, whereas the response is measured at the individual level. As we discuss below, our sample is restricted to households with two working-age members. Hence, when we estimate the income process jointly with the price per calorie, we specify two equations, one for each adult, as:

(5a) $$\Delta u_{1h,t}^{PC}=\phi_{1}^{PC}\eta_{h,t}+\psi_{1}^{PC}\varepsilon_{h,t}+\Delta \xi_{1h,t}^{PC}$$

(5b) $$\Delta u_{2h,t}^{PC}=\phi_{2}^{PC}\eta_{h,t}+\psi_{2}^{PC}\varepsilon_{h,t}+\Delta \xi_{2h,t}^{PC}\text{.}$$

This estimates the effects of permanent and transitory income shocks, *η_h,t_* and *ε_h,t_*, on the price per calorie with gender specific factor loadings $\phi_{1}^{PC}$ and $\psi_{1}^{PC}$ for men, and $\phi_{2}^{PC}$ and $\psi_{2}^{PC}$ for women, allowing us to test whether the response to income shocks differs by gender. Again, the closer the factor loadings are to zero, the better the insurance. Furthermore, we allow the innovations in the price per calorie to be correlated between the two household members: *σ*_*ξ*12,*t*_ ≠ 0, reflecting household level unobservables.

### 3.5 Income and energy intakes

We next estimate the degree of transmission of income shocks to actual individual-level energy intakes. For this, we use a model like equation (5a) and (5b), but replace the outcome variables with $\Delta u_{1h,t}^{C}$ and $\Delta u_{2h,t}^{C}$, where the superscript *C* denotes energy intake (calories). We again allow the innovations in energy intake to be correlated between the two household members: *σ*_*ξ*12,*t*_ ≠ 0. The direction of this correlation is theoretically ambiguous: although one would expect the energy intake of two household members to be positively correlated, there are situations in which we may expect this to be negative. For example, holding income constant, an increase in men’s energy intake may lead to a decrease in women’s energy intake if households are sufficiently income constrained.

In addition to estimating the degree to which income shocks are transmitted to energy intakes, the factor loadings *ϕ* and *ψ* can be used to calculate the extent to which households use additional insurance mechanisms to protect their energy intakes. As *ϕ^E^* and *ϕ^C^* (*ψ^E^* and *ψ^C^* for transitory shocks) are elasticities of consumption and energy intake respectively, $\left(\phi^{E}-\phi^{C}\right)/\phi^{E}$ provides an estimate of the proportion of the effect of income on expenditures that is protected through the various insurance mechanisms available to individuals. Conversely, *ϕ^C^/ϕ^E^* indicates the proportion of the effect of income shocks on food expenditures that is transmitted to energy intakes.

To explore whether there is evidence of differential response to positive and negative income shocks, we use the following two-step process. First, we predict the factor scores of the permanent and transitory income shocks from equation (3), denoted by ${\hat{\eta }}_{ht}$ and ${\hat{\varepsilon }}_{ht}$. Second, we run the following OLS regression:

$$\Delta u_{iht}^{C}=\beta_{0}+\beta_{1}{\hat{\eta }}_{ht}\cdot 1\left[{\hat{\eta }}_{ht}<0\right]+\beta_{2}{\hat{\eta }}_{ht}\cdot 1\left[{\hat{\eta }}_{ht}\geq 0\right]+\beta_{3}{\hat{\varepsilon }}_{ht}\cdot 1\left[{\hat{\varepsilon }}_{ht}<0\right]+\beta_{4}{\hat{\varepsilon }}_{ht}\cdot 1\left[{\hat{\varepsilon }}_{ht}\geq 0\right]+e_{iht}$$

where 1[·] is an indicator function that equals one if the expression between brackets holds, and zero otherwise. The parameters *β*_1_ and *β*_2_ estimate the extent to which households respond to negative and positive *permanent* income shocks respectively, whereas *β*_3_ and *β*_4_ denote the response to negative and positive *transitory* income shocks. The term *e_iht_* denotes the residual. We estimate this not only for individual energy intakes, but also for the aggregate expenditure groups (food, clothes/shoes, and other goods), and the food categories.

### 3.6 Income and dietary composition

Finally, we explore the effects on dietary composition, estimating individuals’ fat and protein intake response to income shocks. For this, we jointly estimate the income process (3) with:

(6a) $$\Delta u_{1h,t}^{F}=\phi_{1}^{F}\eta_{h,t}+\psi_{1}^{F}\varepsilon_{h,t}+\Delta \xi_{1h,t}^{F}$$

(6b) $$\Delta u_{2h,t}^{F}=\phi_{2}^{F}\eta_{h,t}+\psi_{2}^{F}\varepsilon_{h,t}+\Delta \xi_{2h,t}^{F}$$

(6c) $$\Delta u_{1h,t}^{P}=\phi_{1}^{P}\eta_{h,t}+\psi_{1}^{P}\varepsilon_{h,t}+\Delta \xi_{1h,t}^{P}$$

(6d) $$\Delta u_{2h,t}^{P}=\phi_{2}^{P}\eta_{h,t}+\psi_{2}^{P}\varepsilon_{h,t}+\Delta \xi_{2h,t}^{P}\text{,}$$

where the superscripts *F* and *P* refer to fat and protein intake respectively. This allows income shocks to have different effects on men and women’s fat and protein intake. We also allow the innovations to covary across the four equations to account for residual clustering in fat and protein intakes.

## 4 Data

### 4.1 Russia longitudinal monitoring survey

We use the Russia Longitudinal Monitoring Survey (RLMS); a longitudinal study of population dwelling units in the Russian Federation. It includes a static sample of dwellings that are visited in each round of data collection, where all individuals within that dwelling are interviewed. The data were specifically set up to monitor the effects of the Russian reforms. We use the RLMS data from 1994 to 2005, with the exception of 1997 and 1999, when the survey was not administered.^9^ The RLMS comprises 38 primary sampling units (municipalities) that are representative of the whole country. There are approximately 10,000 adults and 4000 households per round of data collection, where the household is defined as all those living together and sharing income and expenses.

The response rate in the RLMS is relatively high, exceeding 80% for households and about 97% for individuals within households. Furthermore, attrition is generally low compared to similar panel surveys in other countries, partly due to lower mobility and infrequent changes of address (Gorodnichenko et al., 2010).

Our sample selection process is as follows. We restrict the data to households with two working-age (age 18–60) members; a man and woman. Hence, our analyses do not include the most vulnerable households, such as single mothers or older people living alone. Although these households are interesting in their own right, the mechanisms available for any nutrition insurance are likely to be different, and with that, so is their response to income shocks. Hence, we focus on a more homogeneous sample – households with two working-age adults, with or without children or senior (60+) members – allowing us to make inferences about a specific population. We explore whether households with children or senior members respond differently to those without in Section 6.1. Nevertheless, our sample restriction means that our results do not necessarily generalise to the full Russian population. Furthermore, as we model changes in income and consumption or diet, we drop households that are observed only once in the observation period. The selection leads to a sample of 3954 adults nested within 1977 households.

Where individuals have missing values on age, education and region, we impute these from observed values in previous waves. Where households and individuals have missing values on income, expenditures, and nutritional intakes, we use multiple multivariate imputation of chained equations (van Buuren, 2012). The chained equations are fitted jointly to the ten years of data, account for the individual- and household-level clustering, and adjust for predictors of missingness. The latter include a quartic polynomial in age and the educational level of the two adult household members, the number of children, and a set of location characteristics. We create five “complete” data sets and estimate our models of interest on each, combining the parameter estimates and standard errors using Rubin’s combination rules (Rubin, 1987) to reflect imputation uncertainty (Carpenter and Kenward, 2013, Schafer, 1997).

Our measure of income is the logarithm of real monthly household disposable income, measured over the 30 days prior to the interview. This includes contractual labour income, any payments in kind, income from selling home-produced goods, net private transfers, financial income from interest and dividends, and government transfers (including state child benefits, unemployment benefits, stipends, and government welfare payments).

We distinguish between the following groups of expenditures: food, clothes/shoes, and other goods (including durables, services, utilities, and fuel).^10^ To create a measure for real monthly household food expenditures, we use information on the quantity and monetary value of the previous week’s purchases on 56 categories of food, alcoholic and non-alcoholic drinks, and tobacco products. Expenditures on services, utilities and fuel are measured in the month prior to the interview, whilst clothes and durables are measured in the three months prior to the interview. We convert all expenditures to monthly values. We also use household expenditures on different food groups, distinguishing between grain, meat, dairy, fruit, sweets, and beverages.^11^ The interviewer was instructed to speak with the person who knows most about the family’s shopping, which typically meant the senior woman in the household. Income and expenditures are measured in the same way across all years, and are deflated to December 2000 prices using the national monthly consumer price index at the date of interview.^12^

The RLMS is one of very few household surveys where much effort is spent on obtaining good measurements on energy intakes at the individual-level. In every wave apart from 1996, trained interviewers conducted a standard 24-h dietary recall of each household members’ food intakes, using colour photos of foods to assist in assessing portion sizes. Individuals report each food item consumed, place of consumption and preparation, method of preparation, and time of consumption. The tables are then translated into energy intake, and the per cent of daily energy obtained from fat and from protein, using food composition tables developed for the Russian diet (Zohoori et al., 2001, Popkin et al., 1996).^13^

### 4.2 Descriptive statistics

We start by presenting the summary statistics in Table B1, Appendix B, showing the data averaged over all years. As commonly shown in the RLMS, combining all food and non-food expenditures exceeds household income (see e.g. Steven and Stillman (2008) and Gorodnichenko et al. (2010), among others). The difference cannot be attributed to dissaving, as most households do not have financial assets. Instead, this is likely to be due to underreporting of income due to a fear of disclosure of individuals’ responses to tax authorities (Gorodnichenko et al., 2010).

Table B1 shows that male and female energy intake is relatively low, suggesting these are underestimates of individuals’ true intake, as is common in 24-h recall data. We assume, however, that the under-reporting does not vary systematically by year, leaving our analyses unaffected. On average, approximately 34% and 13% of individuals’ total energy consumption consists of fats and proteins respectively, with little difference between men and women.

Almost half of the sample consists of households with four or more members, 64% is involved in (at least some) home production of foods, and 70% live in an urban area. Consistent with the World Bank (1995), we find that women, on average, are higher educated than men.

**Fig. 1:**
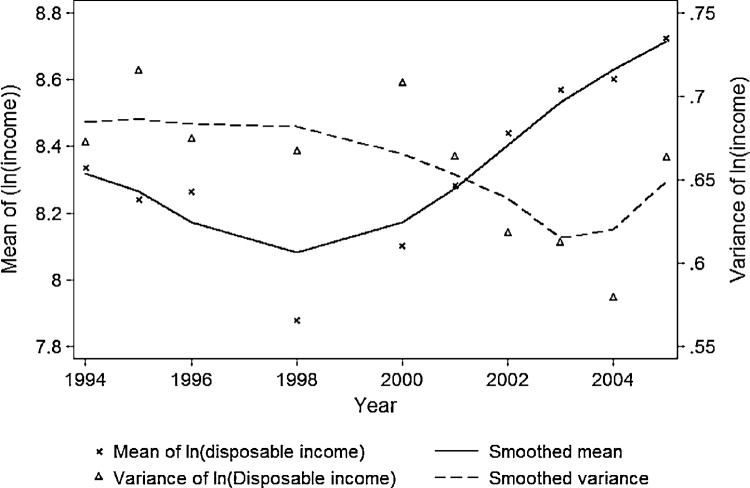
Time-varying mean and variance of log real disposable income. Note: Income is defined as average monthly household disposable income and is in constant December 2000 prices (deflated using national monthly CPI and date of interview).

Fig. 1 plots the means and variances of monthly log real income, where the smoothed lines illustrate the general trends for ease of legibility. Mean income (solid line) drops substantially during the economic downturn and increases during the recovery. The variances (dashed line) are relatively constant during the collapse of the economy, but reduce in the recovery, with a slight rise at the end of the observation period.

**Fig. 2:**
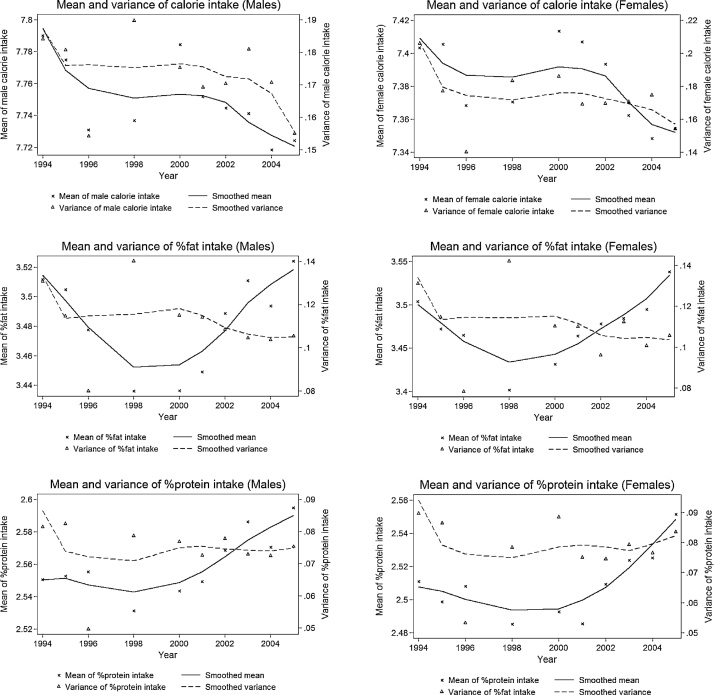
Time-varying mean and variance of individual log energy, fat and protein intakes, by gender.

Fig. 2 presents trends in the means and variances of log energy, fat and protein intakes. The figure shows that mean energy intakes reduce slightly over time for both men and women. Note, however, that our sample ages over time, which is not taken into account in these figures. The mean fat and protein intakes follow clear U-shape trends, suggesting that both fat and protein intakes are positively related to the business cycle, reducing during the downturn and increasing during the recovery.

**Fig. B2:**
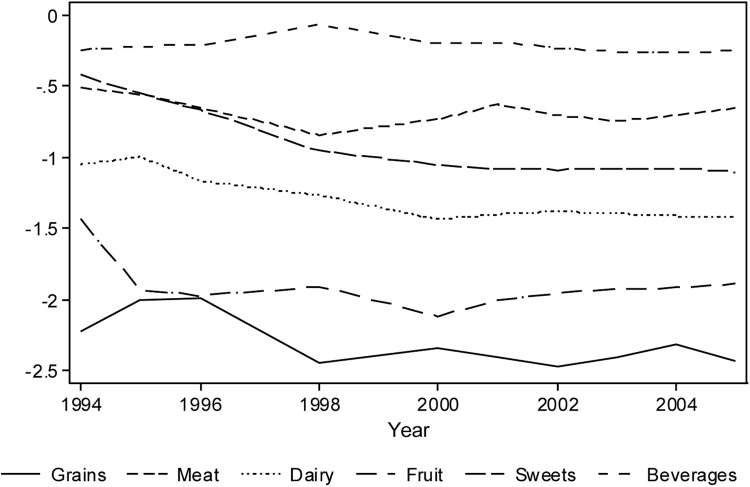
Trends in the natural logarithm of real prices for the main food groups from 1994 to 2005.

Finally, we investigate the role of prices in our analyses. Indeed, if prices change differentially across different goods or regions, this may affect both income and consumption, potentially biasing our estimates. We observe detailed information on the prices of 56 different foods within the six larger food groups we use (grain, meat, dairy, fruit, sweets, and beverages). Eyeballing the mean real price of each food group between 1994 and 2005 in Fig. B2 suggests a slight downward trend. Column 1 of Table B2 confirms this, showing that the real price of grain reduces by an average of 3% per year. There is some evidence of differential price trends for sweets and beverages, relative to grains. In particular, we find that the price of sweets reduces by an additional 3 percentage points per year, whilst the price of beverages remains constant. Although this suggests that there may be some differential trends across the food groups, they are relatively small.

To explore this in more detail, columns 2–9 of Table B2 allow for differential trends within each of the regions. Although the grain price trends differently across regions, reducing by 2–6% each year depending on the region, the other prices show similar trends relative to grains. Note, however, that any regional differences in food prices are accounted for, as we model expenditure net of predictable components (see Equation (2)), which include region dummies.

## 5 Results

### 5.1 Income and expenditures

Table 1 presents the estimates from the joint income-expenditure model of Eqs. (3) and (4), distinguishing between food, clothes/shoes and other spending.^14^ As the estimates of the permanent and transitory income variances are very similar to the income-only model (shown in Appendix B), we do not report these (they are available upon request).

The factor loadings on the permanent income shock (ϕ) suggest that a 10% permanent drop in income induces a 5.7% permanent drop in expenditures on food, a 7.8% drop in expenditures on clothes, and a 10.3% drop in expenditures on other goods. This suggests that households cut back disproportionately on other goods to minimize the reduction in spending on food, consistent with Engel’s (1857) findings that families tend to sacrifice higher-order wants to satisfy more basic wants (i.e. food). The factor loadings on the transitory shocks are relatively small. Specifically, a 10% negative transitory income shock reduces food, clothes/shoes and other spending by 1.6%, 1.4% and 1.7% respectively.^15^

We next examine whether households simply reduce their food expenditures proportionally on each food group, or whether they change the composition of the food consumption basket. We distinguish between the effects of income shocks on expenditures on grains, meats, dairy, fruit and vegetables, sweets and beverages. Table 2 shows that permanent and transitory income shocks have substantially different effects on different food groups. For example, the effect of permanent income shocks on grain expenditures is significantly different from that on meat (p=0.04), dairy (p=0.03), fruit (p<0.001), sweets (p=0.009) and beverages (p=0.01), providing clear evidence that households change the composition of the food basket in response to income shocks. Specifically, expenditures on grains are fully insured against permanent income shocks, with the factor loading *ϕ* not significantly different from zero, whilst they do respond slightly to transitory income shocks. In contrast, spending on other food categories reacts strongly to permanent income shocks, with a 10% drop in income reducing spending on meat, dairy products, fruit and vegetables, sweets, and beverages by between 3.8-6.0%. Hence, in addition to showing the heterogeneity *across* different consumption categories, this demonstrates the importance of allowing for heterogeneity *within* food consumption, which is concealed in analyses that just use the total, aggregated spending (see also Aguiar and Hurst, 2013). This is likely to be especially important in countries such as Russia, where subsistence agriculture plays an important role. Indeed, our data show that the average household produced 331 kg of dairy (milk and eggs) and only 36 kg of meat per year. This suggests that households will be less able to insure their consumption of meat compared to their consumption of dairy.

**Table 2:** Estimates of the joint income-expenditure model, distinguishing by food group.

	(1) Grains	(2) Meat	(3) Dairy	(4) Fruit & Veg	(5) Sweets	(6) Beverages
Permanent income shock *Φ*	0.052	0.557	0.383	0.598	0.520	0.575
	(0.077)	(0.114)	(0.061)	(0.102)	(0.073)	(0.087)

Transitory income shock *ψ*	0.062	0.110	0.152	0.077	0.142	0.156
	(0.030)	(0.062)	(0.042)	(0.067)	(0.038)	(0.054)

*Notes*: Standard errors in parentheses, clustered by household. All estimates are obtained from one model. The table only presents the factor loadings; all variance and covariance estimates are available upon request. The sample includes 1977 households.

### 5.2 Income and the price of calories

To explore whether the composition change in the food basket is driven by individuals trying to protect their energy intake, Column 1 of Table 3 presents the estimates from the joint model of income and the number of calories consumed per Ruble spent on food. This shows that negative income shocks increase the number of calories consumed per Ruble spent, with no significant differences between men and women: a 10% drop in income increases the number of calories per Ruble spent by 4.5% and 4.3% for men and women respectively. This shows a clear reduction in the price of calories when faced with negative permanent income shocks, suggesting that individuals purchase ‘cheaper’ calories when faced with sudden permanent drops in income. However, there is no evidence that transitory income shocks affect the number of calories purchased per Ruble.

**Table 3:** Estimates of the joint income-energy intake model, and income-calorie per Ruble spent on food .

	(1) $ln\left(\frac{energy}{food\;expenditures}\right)$		(2) ln(energy)	
Permanent income shock *Φ*_men_	−0.454	(0.106)	0.067	(0.044)
Transitory income shock *ψ*_men_	−0.113	(0.148)	0.064	(0.019)
Permanent income shock *Φ*_women_	−0.426	(0.100)	0.090	(0.041)
Transitory income shock *ψ*_women_	−0.157	(0.103)	0.026	(0.021)

*Notes*: Standard errors in parentheses, clustered by household. All estimates in each column are obtained from one model. The table only presents the factor loadings; all variance and covariance estimates are available upon request. The sample includes 3954 individuals nested in 1977 households.

### 5.3 Income and energy intake

We next estimate the energy intake response to income shocks. As we observe *actual* intakes, this incorporates any changes due to adjustments of the food consumption basket. Hence, we examine whether the above adjustments (protecting food spending, changing the composition of the food basket, and buying cheaper calories) are sufficient to insure individuals’ energy intakes. If these changes are sufficient to fully insure energy intakes, the factor loadings on the permanent and transitory shocks will not be significantly different from zero.

Table 3, column 2, presents the estimates from the joint income-energy intake model. The factor loadings suggest that a permanent drop in income leads to a reduction in energy intake for both men and women, though only for women is this significantly different from zero at conventional levels. In contrast, transitory income shocks only affect men’s energy intakes, with no significant effect on women’s. Hence, women seem somewhat better protected against transitory income shocks compared to men, but they are less well protected against permanent income shocks. The latter may reflect women acting as the buffer in the household, reducing their own intake when faced with permanent reductions in resources to protect the other family members, as is well known especially in developing countries (see e.g. Holmes et al., 2009). Furthermore, the finding that women are better protected against *transitory* income shocks may reflect the fact that, in general, any additional items of food (e.g. treats, left-overs) tend to be consumed by men, who are more likely to do physical work. However, with temporary negative shocks, these treats must be foregone, and hence men must reduce their consumption whilst women’s intakes remain relatively constant. This, however, is a speculation, and we cannot explore this in more detail. Nevertheless, if we look at the actual effect, it is very small: a 10% permanent drop in income reduces men and women’s energy intake by 0.7% and 0.9%; equivalent to around 5 calories per day. As one pound of body weight is equal to, on average, 3500 calories, this suggests it takes individuals about 700 days to lose one pound of body weight when exposed to a 10% permanent negative income shock. Hence, despite it being significant, the response is minimal.

**Fig. B5:**
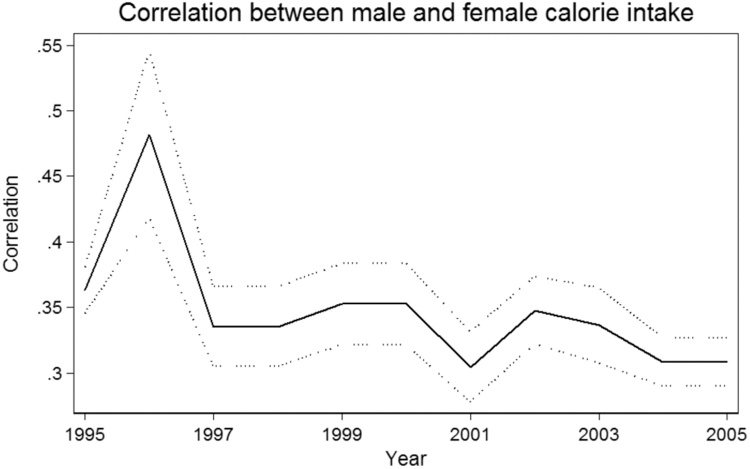
The derived correlations in male-female energy intake.

The male-female covariance in energy intake is always positive (not shown here), suggesting that where one individual has a higher-than-predicted energy intake, so does their partner. The corresponding correlations, presented in Appendix B, Fig. B5, are around 0.35 at the start of the observation period, decreasing to around 0.3, but with a spike in 1996.

The above findings allow us to look more closely at *the extent to which* energy intake is insured against income shocks. Indeed, comparing the relative magnitudes of the transmission parameters for food expenditures to those for energy intakes, we find that permanent income shocks affect both, but that energy intakes are better insured than food expenditures. Specifically, a 10% permanent income shock changes food expenditures by 5.7% (Table 1), whereas it changes energy intake by 0.7–0.9% (Table 3). Put differently, 12–16% $\left(\frac{0.7}{5.7}-\frac{0.9}{5.7}\right)$ of the effect of permanent income shocks on food expenditures is transmitted to changes in energy intakes, with 84–88% insured through the various insurance mechanisms available to households.

**Table 1:** Estimates of the joint income-expenditure model, distinguishing by expenditure category.

	(1) Food		(2) Clothes/shoes		(3) Other	
Permanent income shock *Φ*	0.572	(0.112)	0.782	(0.120)	1.026	(0.125)
Transitory income shock *ψ*	0.161	(0.050)	0.142	(0.035)	0.170	(0.047)

*Notes*: Other expenditures include spending on durables, fuel, utilities and services. Standard errors in parentheses, clustered by household. All estimates are obtained from one model. The table only presents the factor loadings; all variances and covariances are available upon request. Sample includes 1977 households.

### 5.4 Positive and negative income shocks

Table 4, Table 5, Table 6 distinguish between the effects of positive and negative income shocks on the aggregated spending categories, the different food groups, and individual energy intake respectively. Table 4 shows that households tend to respond more to negative compared to positive permanent income shocks for the aggregated spending categories. For example, a 10% negative permanent income shock reduces spending on food by 5.2%, whilst a similar-sized positive shock increases food spending by only 3.3%. This suggests that negative permanent income shocks have larger effects on household spending that positive shocks. We find a similar pattern for spending on grains, meat, dairy and fruit and vegetables, as shown in Table 5. Conversely, however, we find the opposite for transitory shocks: negative transitory shocks have smaller effects on consumption than positive transitory shocks; this finding is consistent across the aggregated spending categories (Table 4), as well as the different food groups (Table 5). This suggests that households respond more to permanent *reductions* than to permanent *rises* in income. Similarly, households use temporary gains in income to buy more (or more luxurious) goods, but do not respond as such to transitory reductions in income.

**Table 4:** Positive and negative income shocks: aggregate spending categories.

		(1) Food		(2) Clothes/shoes		(3) Other	
Negative permanent shocks	${\hat{\eta }}_{ht}\cdot 1\left[{\hat{\eta }}_{ht}<0\right]$	0.521	(0.123)	0.927	(0.267)	1.160	(0.252)
Positive permanent shocks	${\hat{\eta }}_{ht}\cdot 1\left[{\hat{\eta }}_{ht}\geq 0\right]$	0.328	(0.200)	0.648	(0.333)	1.201	(0.218)
Negative transitory shocks	${\hat{\varepsilon }}_{ht}\cdot 1\left[{\hat{\varepsilon }}_{ht}<0\right]$	0.058	(0.029)	0.129	(0.069)	0.121	(0.071)
Positive transitory shocks	${\hat{\varepsilon }}_{ht}\cdot 1\left[{\hat{\varepsilon }}_{ht}\geq 0\right]$	0.147	(0.033)	0.142	(0.072)	0.219	(0.067)

*Notes*: The estimates are obtained from the two-step procedure described in Section 3.3. Standard errors in parentheses, clustered by household. Other expenditures include spending on durables, fuel, utilities and services. The sample includes 1977 households.

**Table 5:** Positive and negative income shocks: spending on different food groups.

		(1) Grains		(2) Meat		(3) Dairy		(4) Fruit & veg		(5) Sweets		(6) Beverages	
Negative permanent shocks	${\hat{\eta }}_{ht}\cdot 1\left[{\hat{\eta }}_{ht}<0\right]$	0.191	(0.126)	0.506	(0.208)	0.399	(0.143)	0.540	(0.354)	0.644	(0.208)	0.457	(0.214)
Positive permanent shocks	${\hat{\eta }}_{ht}\cdot 1\left[{\hat{\eta }}_{ht}\geq 0\right]$	0.071	(0.179)	0.346	(0.320)	0.075	(0.265)	0.308	(0.225)	0.684	(0.272)	0.489	(0.233)
Negative transitory shocks	${\hat{\varepsilon }}_{ht}\cdot 1\left[{\hat{\varepsilon }}_{ht}<0\right]$	0.024	(0.042)	0.073	(0.050)	0.079	(0.041)	0.098	(0.118)	0.016	(0.076)	0.116	(0.053)
Positive transitory shocks	${\hat{\varepsilon }}_{ht}\cdot 1\left[{\hat{\varepsilon }}_{ht}\geq 0\right]$	0.088	(0.053)	0.095	(0.053)	0.149	(0.069)	0.165	(0.067)	0.032	(0.079)	0.158	(0.069)

*Notes*: The estimates are obtained from the two-step procedure described in Section 3.3. Standard errors in parentheses, clustered by household. The sample includes 1977 households.

**Table 6:** Positive and negative income shocks: energy (kcal) per Ruble and total energy.

		Men		Women	
Panel A: Energy (kcal) per Ruble					
Negative permanent shocks	${\hat{\eta }}_{ht}\cdot 1\left[{\hat{\eta }}_{ht}<0\right]$	−0.414	(0.133)	−0.457	(0.152)
Positive permanent shocks	${\hat{\eta }}_{ht}\cdot 1\left[{\hat{\eta }}_{ht}\geq 0\right]$	−0.265	(0.199)	−0.238	(0.190)
Negative transitory shocks	${\hat{\varepsilon }}_{ht}\cdot 1\left[{\hat{\varepsilon }}_{ht}<0\right]$	−0.022	(0.039)	−0.013	(0.035)
Positive transitory shocks	${\hat{\varepsilon }}_{ht}\cdot 1\left[{\hat{\varepsilon }}_{ht}\geq 0\right]$	−0.099	(0.042)	−0.128	(0.044)

Panel B: Total energy					
Negative permanent shocks	${\hat{\eta }}_{ht}\cdot 1\left[{\hat{\eta }}_{ht}<0\right]$	0.107	(0.060)	0.057	(0.068)
Positive permanent shocks	${\hat{\eta }}_{ht}\cdot 1\left[{\hat{\eta }}_{ht}\geq 0\right]$	0.086	(0.093)	0.106	(0.070)
Negative transitory shocks	${\hat{\varepsilon }}_{ht}\cdot 1\left[{\hat{\varepsilon }}_{ht}<0\right]$	0.038	(0.023)	0.044	(0.026)
Positive transitory shocks	${\hat{\varepsilon }}_{ht}\cdot 1\left[{\hat{\varepsilon }}_{ht}\geq 0\right]$	0.052	(0.018)	0.019	(0.019)

*Notes*: The estimates are obtained from the two-step procedure described in Section 3.3. Standard errors in parentheses, clustered by household. The sample includes 3954 individuals, nested in 1977 households.

Table 6 shows that the effects of positive shocks on both the number of calories per Ruble spent on food and on total energy intake do not differ from the effects of negative shocks. Focusing first on the number of calories consumed per Ruble spent on food, presented in Panel A, we find that the response to negative permanent income shocks is larger for both men and women, though not significantly so, than the response to positive permanent income shocks (p=0.61 and *p *= 0.46 for men and women respectively). In other words, individuals increase the calories per Ruble spent on food in response to a negative shock by more than the reduction in response to a positive shock. We find the opposite, however, for transitory shocks, where both men and women significantly reduce the number of calories per Ruble spent on food in response to a positive transitory shock, but show no significant response to a negative transitory shock.

Looking at the total energy consumed, in Panel B, we find that men respond slightly more, though not significantly so, to negative compared to positive permanent shocks (a test that the two are equal gives p=0.86): a 10% negative permanent income shock reduces men’s energy intake by 1.1%, whilst a similar-sized positive shock increases their intake by 0.9%. We find the opposite for women. The coefficient on transitory shocks suggests that women reduce their energy intakes in response to a negative shock, whilst they react less to positive shocks; consistent with the theory for credit constrained households (see Section 3.1).

### 5.5 Income and nutritional composition

Finally, we explore the extent to which income shocks affect the nutritional composition, looking at individuals’ fat and protein intakes as a proportion of their total energy intake. Table 7 reports the factor loadings, showing that permanent income shocks significantly affect both fat and protein intakes for men and women, with no effects for transitory income shocks. The estimates suggest that a 10% permanent drop in income reduces male fat and protein intake by 1.1% and 1.0% respectively, whilst it reduces female fat and protein intakes by 0.9% and 0.7%. However, we do not find significant differences in the response of fat intakes to income shocks compared to that of protein intakes (p=0.78 and *p =* 0.50 for men and women respectively).

**Table 7:** Estimates of the joint income, fat and protein intake model .

	Fat		Protein	
Permanent income shock *Φ*_men_	0.107	(0.042)	0.098	(0.021)
Transitory income shock *ψ*_men_	0.008	(0.010)	0.001	(0.014)
Permanent income shock *Φ*_women_	0.090	(0.027)	0.071	(0.016)
Transitory income shock *ψ*_women_	0.020	(0.012)	−0.006	(0.011)

*Notes*: Standard errors in parentheses, clustered by household. All estimates are obtained from one model. The table only presents the factor loadings for men and women; all variance and covariance estimates are available upon request. The sample includes 3954 individuals nested in 1977 households.

**Fig. B6:**
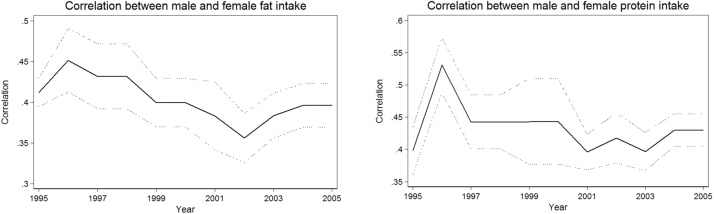
The derived correlations in male-female fat and protein intakes.

Fig. B6, Appendix B, presents the derived correlations between male and female fat and protein intakes. These are around 0.4 and relatively constant over the observation period. This implies that the correlation of diets within the household does not respond much to changes in economic conditions.

## 6 Robustness analysis

### 6.1 Differential effects pre- and post-crisis

As discussed in Section 2, the economic crisis in Russia can be separated into two periods: the downturn pre-1998 and the recovery thereafter. Our previous analyses estimate one factor loading for each of permanent and transitory income shocks for men and women. However, one may expect the response to income shocks to be different *during* as opposed to *after* the height of the crisis. We therefore next explore whether there are differences in the factor loadings for male and female energy intake before and after 1998.

Table 8 presents the results. Although these are generally less precisely estimated, they suggest that men do not change their energy intakes in response to permanent income shocks during the height of the crisis, whereas women are much less protected: a 10% permanent drop in income reduces men’s energy intake by 0.06%, but it reduces women’s energy intake by 1.4%. This is consistent with the idea that women may act as buffers in the household, reducing their own intake when faced with permanent reductions in resources to protect the other family members. During the recovery phase, the response for men is slightly larger than that for women. However, in both periods, the estimates are not significantly different from zero, nor from each other. Hence, we cannot make strong statements regarding the differential energy intake response during the downturn and recovery.

**Table 8:** Estimates of the joint income-energy intake model, allowing for different factor loadings pre- and post-crisis .

	ln(energy)	
Permanent income shock, pre-1998 *Φ*_men_	0.006	(0.121)
Permanent income shock, post-1998 *Φ*_men_	0.093	(0.060)
Transitory income shock, pre-1998 *ψ*_men_	0.064	(0.026)
Transitory income shock, post-1998 *ψ*_men_	0.078	(0.051)
Permanent income shock, pre-1998 *Φ*_women_	0.142	(0.125)
Permanent income shock, post-1998 *Φ*_women_	0.056	(0.047)
Transitory income shock, pre-1998 *ψ*_women_	0.016	(0.028)
Transitory income shock, post-1998 *ψ*_women_	0.005	(0.041)

*Notes*: Standard errors in parentheses, clustered by household. All estimates are obtained from one model. The table only presents the factor loadings; all variance and covariance estimates are available upon request. The sample includes 3954 individuals nested in 1977 households.

### 6.2 Subgroup analysis

To examine whether different types of households are differentially affected by income shocks, we explore whether the estimates for energy intake differ across different subgroups, distinguishing between households with and without children, with and without seniors, between those involved in home production or not, between higher and lower educated households, between households with few (≤1) versus more (≥2) assets, and between households that indicate to have given or received money/goods from an informal network in the past 30 days.^16^

The extent to which the different subgroups can insure their consumption is ambiguous. For example, it is common in Russia for households to include more than two generations. On the one hand, grandparents may act as a buffer against potential income uncertainty. On the other, having to feed and protect all members’ nutritional intakes may be more difficult with an older generation in the household. Similarly, whether high or low educated households differ in the extent to which they can insure their consumption depends on the relative importance of the insurance mechanisms for these groups. Although subsistence agriculture plays a role in both poor and affluent families, the poor may benefit from this more, increasing their insurance compared to the more affluent families. We explore this empirically.

By analysing different subgroups, the parameters become less precisely estimated, making it difficult to distinguish between the different estimates. However, the general patterns in Table 9 suggest that men with children respond more to transitory income shocks compared to men without children, with no large differences for women. A larger permanent income factor loading for men in households with senior members indicates less insurance compared to households without senior members. Similarly, there is some suggestion that men involved in home production are better insured than those not doing any home production. Furthermore, the estimates suggest that higher educated men and those with more assets are slightly better insured compared to lower educated men and those with fewer assets. Finally, the estimates suggest that men in households that used an informal network in the past 30 days are slightly better insured against permanent income shocks than men in households that did not use such a network. In general, this suggests some differential insurance between different types of households, but with relatively large standard errors, we cannot statistically distinguish between the different groups.

**Table 9:** Subgroup analysis of the joint income-energy intake model.

	With or without children		With or without senior household members		Home production		By education		By asset-indicator		With or without informal network	
	(1) Without	(2) With	(3) Without senior	(4) With senior	(5) No home production	(6) Home production	(7) ≤Secondary education	(8) > Secondary education	(9) ≤1 asset (out of 4)	(10) ≥2 assets (out of 4)	(11) Without	(12) With
Permanent income shock *Φ*_men_	0.068	0.063	0.052	0.087	0.075	0.057	0.073	0.056	0.099	0.057	0.094	0.056
	(0.085)	(0.041)	(0.047)	(0.046)	(0.067)	(0.039)	(0.059)	(0.039)	(0.075)	(0.044)	(0.075)	(0.038)

Transitory income shock *ψ*_men_	0.008	0.070	0.064	0.063	0.078	0.057	0.076	0.050	0.040	0.070	0.052	0.069
	(0.069)	(0.021)	(0.022)	(0.039)	(0.028)	(0.026)	(0.022)	(0.031)	(0.044)	(0.020)	(0.036)	(0.020)

Permanent income shock *Φ*_women_	0.103	0.082	0.091	0.078	0.067	0.098	0.078	0.095	0.101	0.083	0.061	0.095
	(0.078)	(0.042)	(0.048)	(0.045)	(0.060)	(0.041)	(0.050)	(0.054)	(0.067)	(0.043)	(0.069)	(0.041)

Transitory income shock *ψ*_women_	0.014	0.027	0.031	0.015	0.051	0.015	0.025	0.033	0.016	0.031	0.040	0.023
	(0.086)	(0.020)	(0.020)	(0.054)	(0.029)	(0.027)	(0.031)	(0.025)	(0.048)	(0.024)	(0.027)	(0.026)

*Notes*: Standard errors in parentheses, clustered by household. All estimates are obtained from one model. The table only presents the factor loadings; all variance and covariance estimates are available upon request. The sample includes 1977 households.

### 6.3 Expenditure decomposition

The above analyses extract the permanent and transitory income shocks directly from the income data. To examine the robustness of these results, we identify the shocks from the expenditure data and estimate their effects on energy and nutritional intakes. Similar to the income-only model, the expenditure-only model captures the expenditure process well. We again find the transitory expenditure shocks to have a higher variance than the permanent expenditure shocks, with the latter remaining relatively stable over the observation period (results available upon request).

As the joint models of expenditure and energy intake or dietary composition estimate the response to a change in *expenditures* rather than *income*, the factor loadings tend to be slightly larger than those presented above (results not shown, but available on request). For example, a 10% permanent expenditure shock reduces energy intake by 1.3% and 1.4% for men and women respectively (compared to 0.7% and 0.9% for income shocks; see Table 3). Generally speaking, however, the analyses that identify shocks from the expenditure data leads to similar findings to those identifying shocks from the income data.

## 7 Discussion and relation to the wider literature

Research exploring the effects of income shocks on expenditures, health and nutrition is not new. Indeed, there has been much work on consumption smoothing in general (for the literature on Russia, see e.g. Skoufias, 2003, Stillman and Thomas, 2008), the effects of income shocks on health outcomes (e.g. Adda et al., 2009, Staudigel, 2016), on food versus non-food spending (e.g. Stillman, 2001; Steven, 2001; Skoufias, 2003), on expenditures, quantities and unit values of foods (e.g. Stillman and Thomas, 2008; Staudigel, 2012), as well as on the nutritional composition of household diets (e.g. Stillman and Thomas, 2008; Burggraf et al., 2015). Others have explored differential effects by subgroups (e.g. Notten and de Crombrugghe, 2012), and differences between positive and negative income shocks (Stillman and Thomas, 2008; Notten and de Crombrugghe, 2012).

Many different methods have been used to estimate these effects, such as fixed and random effect models, error correction models, and demand systems. Furthermore, studies use different data sources, samples, and time periods. Nevertheless, their findings are relatively consistent. In general, the research suggests that households sacrifice non-foods to protect food spending and ensure sufficient nutrients, potentially leading to changes in the food composition basket.

Our research is most similar to that by Stillman and Thomas (2008). Using the RLMS, they find that energy intake is very resilient to ‘short-term’, but less to ‘longer-run’, variations in household resources. Hence, our results are generally consistent with their analyses. However, the identification strategies, as well as the actual parameters that are estimated are very different. First, our focus is on the effect of permanent and transitory income *shocks*, identified from assumptions about the income process, as opposed to the long- and short-run income *components* estimated in Stillman and Thomas (2008), proxied by the average household income over the observation period, and deviations from this mean, respectively. Second, the analyses in Stillman and Thomas (2008) necessarily focuses more on the short-run component, as the long-run component cannot be estimated in a fixed effects model. In effect, our model allows us to decompose their short-run component into a permanent and transitory shock. Our identification strategy allows us to estimate both shocks and compare their magnitudes in all specifications. In fact, our findings suggest that the largest effects are for permanent income shocks, with transitory shocks being much less important for spending and nutritional intakes. Despite this conceptual difference in the estimation approach and methodology, our estimates of the effects of permanent income shocks are similar to those in the existing literature (see e.g. Stillman and Thomas, 2008; Staudigel, 2012).

Furthermore, our research adds to the literature in four main ways. First, due to our rich data and unique context, we are able to use the same sample of individuals over time, and describe links between some of the above sub-questions in more detail. For example, although we find significant changes in response to income shocks in aggregate food spending and in the allocation of spending across different food groups, the effects on macronutrients are small. Hence, despite substantial changes in the food consumption basket, total energy intakes as well as the nutritional composition of the diet remains relatively stable, suggesting that although households reallocate their spending within and across food groups, they maintain the nutritional balance of the diet.

Second, although our methodology has been used to estimate the extent to which households can smooth consumption, it has not been applied to explore the effects of income shocks on nutritional intakes and dietary composition. In addition to estimating the parameters of interest, the empirical approach simultaneously allows the innovations in income and nutritional intakes to covary between the two genders and across different equations (e.g. macronutrients), as well as estimate the correlations between energy intakes within the household. Indeed, our estimates show a positive correlation between male and female energy intakes of around 0.3, suggesting that households are not so constrained that they have to reduce one member’s energy intake when increasing the other’s. Furthermore, our results suggest that the correlation of macronutrient intakes within the household does not respond to changes in economic circumstances. Jointly estimating the effects on men and women also allows us to test whether their response is statistically different from each other. Our results suggest that the energy intake response to permanent shocks does not differ by gender, but we do find that men have a significantly larger response to transitory shocks compared to women.

Third, although we find that permanent income shocks have larger effects than transitory shocks, our results suggest that households respond differently depending on whether these shocks are positive or negative. Indeed, within transitory shocks, negative ones have smaller effects than positive ones, suggesting that households use temporary gains in income to buy more (or more luxurious) goods, but do not respond as such to transitory reductions in income. We do not find this for permanent income, where negative shocks tend to have larger effects on household spending that positive ones.

Finally, our findings allow us to estimate the proportion of energy that is insured against income shocks. We find that this is substantial: between 84 and 88% of energy intakes is protected through the various insurance mechanisms available to households.

In terms of policy implications, our findings are consistent with the existing literature, suggesting that households are able to adapt very well to new and volatile environments. Aguiar and Hurst (2005) show that households smooth consumption in response to *expected* income shocks (i.e. retirement). Griffith et al. (2015) find that households smooth energy and nutritional quality in response to *unexpected* income shocks. The latter, however, follows households in the United Kingdom, where there exists an arguably strong safety net, providing protection to household income. Our study shows that, even in the *absence* of a welfare state or safety net, with large groups of the population living on the poverty threshold, households respond to large unexpected income shocks by reallocating their spending both between and within food groups to ensure relatively constant energy intakes and distributions of macronutrients, leaving the nutritional composition of the diet quite stable.

## 8 Conclusion

The effect of changes in economic circumstances and household income on individuals’ diet and nutritional intakes is a topic that has recently received substantial interest. Evidence from recent recessions show they change household shopping behaviour and food intakes. Understanding these relationships is crucial in the design and evaluation of social insurance and income maintenance policies.

We examine *the extent to which*, as well as *how* individuals ‘insure’ their energy intake in the face of unanticipated shocks to household income. Distinguishing between the effect of permanent and transitory shocks and allowing for partial insurance, our findings suggest that households cut back disproportionately on non-foods when faced with negative income shocks to protect their spending on foods. In addition, we find that households change the composition of their food consumption basket in response to income shocks, with some food groups being fully insured against permanent shocks, and others being only partially insured. This shows the importance of allowing for heterogeneity *across*, as well as *within* different consumption categories. Indeed, we show that the use of disaggregated spending categories provides additional information that is concealed in analyses that just use the total, aggregated, spending. Furthermore, we find that households substitute to cheaper calories when faced with negative income shocks.

Taken together, we show that the vast majority of energy intakes are insured against income shocks. We find that 12–16% of the effect of permanent income shocks on food expenditures is transmitted to changes in energy intakes, with 84–88% insured through the various insurance mechanisms available to individuals. Nevertheless, the changes seen in dietary composition, such as the reductions in meat and dairy with no response in grain consumption, suggests that the economic reforms have in fact *improved* the average Russian diet. This is consistent with other literature (see e.g. FAO, 2003), arguing that the Russian diet has become healthier since the 1990s due to decreases in milk, meat and animal fat consumption, and a rising share of starchy staples like bread and potatoes.

Nevertheless, quantifying the effects of income shocks in terms of calories and body weight, the estimated reduction of 5 calories per day associated with a 10% negative permanent income shock is predicted to lead to a 1 pound reduction in body weight after almost 2 years. Given the high levels of obesity in Russia, this reflects just a minor improvement in the health of the population.

Hence, the results suggest that households are able to smooth their energy intakes substantially, even during periods characterised by substantial economic volatility. We find that households are able to keep their dietary intakes constant when faced with transitory shocks to income, and that they are able to substantially smooth their intakes against permanent shocks, substituting non-food with food expenditures, changing the composition of the food consumption basket, and turning to ‘cheaper’ calories when faced with sudden reductions in income.

## Figures and Tables

**Figure:**
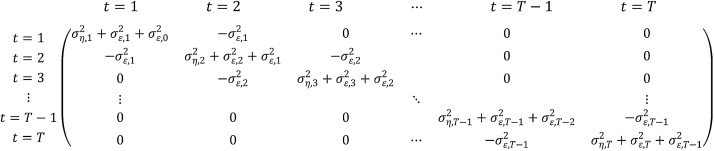


**Fig. B3:**
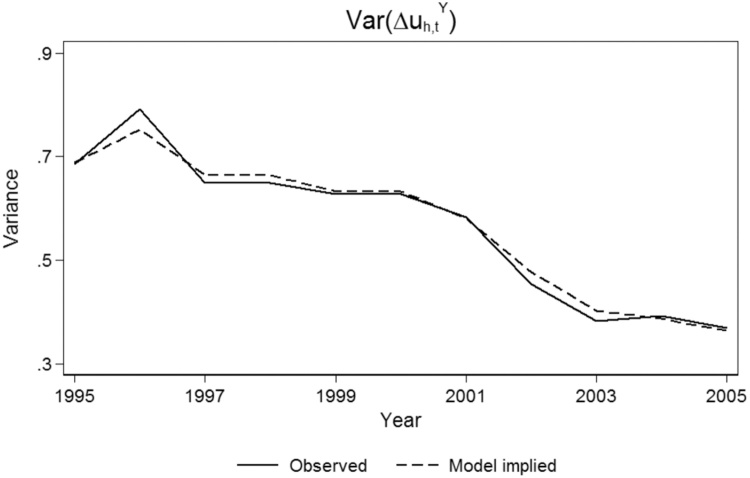
Goodness of fit – comparing the observed and model-implied variance in income growth. Note: Values for 1998 and 2000 are adjusted for the fact that the permanent component is accumulated over two years (see Appendix A). For the years 1997 and 1999, the values are set equal to 1998 and 2000 respectively.

**Table B1:** Household- and individual-level descriptive statistics.

	Mean	Std. dev.
Household-level variables		
Disposable income	5924	(5862)
Total expenditures on food	3309	(3179)
Expenditures on grain	389	(375)
Expenditures on meat	1075	(1460)
Expenditures on dairy	574	(707)
Expenditures on fruit	556	(1229)
Expenditures on sweets	418	(741)
Expenditures on drinks	511	(853)
Expenditures on clothes/shoes	1219	(2738)
Expenditures on other items	3101	(9440)

Individual-level variables		
Male energy intakes	2527	(1076)
Female energy intakes	1749	(739)
Male percent of energy from fat	34.4	(11.3)
Female percent of energy from fat	34.0	(11.2)
Male percent of energy from protein	13.4	(3.71)
Female percent of energy from protein	12.8	(3.74)

Covariates		
Household members: 2	0.16	(0.36)
Household members: 3	0.35	(0.48)
Household members: 4	0.33	(0.47)
Household members: 5	0.16	(0.36)
Any home production	0.64	(0.48)
Urban	0.70	(0.46)
Region: Moscow/St. Petersburg	0.13	(0.34)
Region: North west	0.07	(0.25)
Region: Central	0.17	(0.37)
Region: Volga	0.16	(0.37)
Region: North Caucasus	0.12	(0.32)
Region: Ural	0.15	(0.36)
Region: Western Siberia	0.11	(0.31)
Region: Eastern Siberia	0.00	(0.00)
Age Male	39.62	(8.86)
Age Female	38.78	(8.95)
Male education: Vocational	0.14	(0.35)
Male education: <Secondary	0.00	(0.00)
Male education: Secondary	0.31	(0.46)
Male education: Technical	0.23	(0.42)
Male education: University	0.26	(0.44)
Female education: Vocational	0.07	(0.25)
Female education: <Secondary	0.04	(0.21)
Female education: Secondary	0.20	(0.40)
Female education: Technical	0.42	(0.49)
Female education: University	0.27	(0.44)
Number of individuals	3266	
Number of households	1633	

*Notes*: All income and expenditures (in Rubles) are in constant December 2000 prices (deflated using national monthly CPI and date of interview).

**Table B2:** Trends in real prices from 1994 to 2005, distinguishing by food group.

	(1) All regions	(2) Moscow/St Petersburg	(3) North west	(4) Central	(5) Volga	(6) North Caucasus	(7) Ural	(8) West Siberia	(9) East Siberia
Meat	1.49***	1.47***	1.53***	1.57***	1.43***	1.52***	1.41***	1.51***	1.46***
	(0.11)	(0.10)	(0.11)	(0.10)	(0.12)	(0.13)	(0.11)	(0.12)	(0.12)

Dairy	1.05***	1.11***	1.14***	1.03***	1.02***	1.15***	0.99***	1.01***	1.03***
	(0.11)	(0.10)	(0.11)	(0.10)	(0.12)	(0.13)	(0.11)	(0.12)	(0.12)
	
Fruit and vegetables	0.31	0.20	0.42***	0.31**	0.33**	0.07	0.30*	0.34**	0.37**
	(0.11)	(0.10)	(0.11)	(0.10)	(0.12)	(0.13)	(0.11)	(0.12)	(0.12)

Sweets	1.59***	1.55***	1.51***	1.59***	1.58***	1.72***	1.55***	1.68***	1.52***
	(0.11)	(0.10)	(0.11)	(0.10)	(0.12)	(0.13)	(0.11)	(0.12)	(0.12)

Beverages	1.90***	1.88***	1.79***	1.97***	1.94***	2.04***	1.85***	1.85***	1.73***
	(0.11)	(0.10)	(0.11)	(0.10)	(0.12)	(0.13)	(0.11)	(0.12)	(0.12)

Trend (year)	−0.03***	−0.02*	−0.02*	−0.02*	−0.03*	−0.02	−0.04***	−0.06***	−0.06***
	(0.01)	(0.01)	(0.01)	(0.01)	(0.01)	(0.01)	(0.01)	(0.01)	(0.01)

Trend × Meat	0.02	0.02	0.00	0.01	0.03	0.02	0.03	0.03	0.02
	(0.03)	(0.01)	(0.01)	(0.01)	(0.02)	(0.02)	(0.01)	(0.02)	(0.02)

Trend × Dairy	−0.01	−0.01	−0.03	−0.00	−0.01	−0.01	−0.00	0.00	−0.00
	(0.01)	(0.01)	(0.01)	(0.01)	(0.02)	(0.02)	(0.01)	(0.02)	(0.02)

Trend × Fruit & veg	0.01	0.02	−0.00	0.01	−0.00	0.01	0.02	0.03	0.02
	(0.01)	(0.01)	(0.01)	(0.01)	(0.02)	(0.02)	(0.01)	(0.02)	(0.02)

Trend × Sweets	−0.03*	−0.02	−0.03	−0.03*	−0.04*	−0.04*	−0.02	−0.03*	−0.02
	(0.01)	(0.01)	(0.01)	(0.01)	(0.02)	(0.02)	(0.01)	(0.02)	(0.02)

Trend × Beverages	0.03*	0.03*	0.02	0.03*	0.02	0.03	0.02	0.04*	0.04*
	(0.01)	(0.01)	(0.01)	(0.01)	(0.02)	(0.02)	(0.01)	(0.02)	(0.02)

Intercept	−2.08	−1.98***	−1.98***	−2.18***	−2.19***	−2.30***	−2.09***	−1.91***	−1.84***
	(0.07)	(0.07)	(0.08)	(0.07)	(0.08)	(0.09)	(0.08)	(0.08)	(0.09)

No. of group-years	60	60	60	60	60	60	60	60	60

*Notes*: Real food prices (logarithms) for each food group are collapsed to the food group-year level (column 1) and the food group-year-region level (columns 2–9); Standard errors in parentheses; Reference food group is grains. Year runs from 1 (=1994) to 12 (=2005).

* p < 0.10.

** p < 0.05.

*** p < 0.01.

**Fig. B4:**
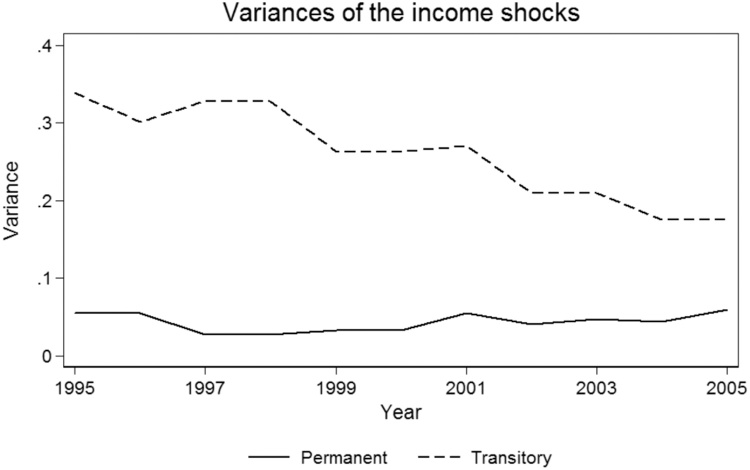
The permanent and transitory income shocks. Note: Values for 1998 and 2000 are adjusted for the fact that the permanent component is accumulated over two years (see Appendix A). For the years 1997 and 1999, the values for the permanent and transitory shocks are set equal to the values in 1998 and 2000 respectively.

## Appendix A Identification

Writing out the income-process for our observation period, we get:

$$\begin{array}{c} \Delta u_{h,1995}^{Y}=\eta_{h,1995}+\Delta \varepsilon_{h,1995}\\ \Delta u_{h,1996}^{Y}=\eta_{h,1996}+\Delta \varepsilon_{h,1996}\\ \begin{array}{c} \begin{array}{c} \vdots \end{array}\\ \Delta u_{h,2005}^{Y}=\eta_{h,2005}+\Delta \varepsilon_{h,2005}\text{,} \end{array} \end{array}$$

which we estimate jointly as a structural equation model. We derive the income covariance matrix based on the assumptions specified in Section 3.2. With $\sigma_{\eta ,t}^{2}$ and $\sigma_{\varepsilon ,t}^{2}$ denoting the period *t* variances of the permanent and transitory income shocks respectively, we can write the elements of this matrix as:

(A.1) $$\text{cov}\left(\Delta u_{h,t}^{Y},\Delta u_{h,t}^{Y}\right)=\sigma_{\eta ,t}^{2}+\sigma_{\varepsilon ,t}^{2}+\sigma_{\varepsilon ,t-1}^{2}$$

(A.2) $$\text{cov}\left(\Delta u_{h,t}^{Y},\Delta u_{h,t-1}^{Y}\right)=-\sigma_{\varepsilon ,t-1}^{2}$$

and zero otherwise, giving:

There are two identification issues. First, $\sigma_{\eta ,1}^{2}$ is not separately identified from $\sigma_{\varepsilon ,0}^{2}$. And second, $\sigma_{\eta ,T}^{2}$ is not separately identified from $\sigma_{\varepsilon ,T}^{2}$. We follow common practice by imposing $\sigma_{\varepsilon ,1}^{2}=\sigma_{\varepsilon ,0}^{2}$, and $\sigma_{\varepsilon ,T}^{2}=\sigma_{\varepsilon ,T-1}^{2}$ (see e.g. Gorodnichenko et al., 2010). We therefore estimate the following parameter vector: $\Lambda^{Y}=\left(\sigma_{\varepsilon ,1}^{2},\sigma_{\varepsilon ,2}^{2},\ldots ,\sigma_{\varepsilon ,T-1}^{2},\sigma_{\eta ,1}^{2},\sigma_{\eta ,2}^{2},\ldots ,\sigma_{\eta ,T}^{2}\right)$, where the superscript Y denotes that the vector refers to the income process.

Note that equation (A.1) and (A.2) do not hold for the years 1998 and 2000, as data were not collected in 1997 or 1999. This implies that we observe the growth in unexplained income over two years in 1998 and 2000. Omitting the h subscript, we can write this as:

$$u_{t}^{Y}-u_{t-2}^{Y}=\left\{\left(P_{t-2}+\eta_{t-1}\right)+\eta_{t}+\varepsilon_{t}\right\}-\left(P_{t-2}+\varepsilon_{t-2}\right)=\eta_{t}+\eta_{t-1}+\varepsilon_{t}-\varepsilon_{t-2}.$$

The corresponding covariance is given by:

$$\text{cov}\left(\Delta u_{h,t}^{Y},\Delta u_{h,t}^{Y}\right)=\sigma_{\eta ,t}^{2}+\sigma_{\eta ,t-1}^{2}+\sigma_{\varepsilon ,t}^{2}+\sigma_{\varepsilon ,t-2}^{2}\text{.}$$

Hence, the permanent income shock is accumulated over two years, and the model-implied permanent variance is the sum of the two components. We deal with this by dividing the estimate for $\sigma_{\eta ,t}^{2}$ by two for the years 1998 and 2000.

To estimate the multivariate response model of household income and expenditures on food, clothes and other goods, we exploit the (auto)covariances of their growth rates. More specifically, in addition to estimating the income process above, we jointly estimate the following expenditure equations (each for food, clothes and other goods):

$$\begin{array}{c} \Delta u_{h,1995}^{E}=\phi^{E}\eta_{h,1995}+\psi^{E}\varepsilon_{h,1995}+\Delta \xi_{h,1995}^{E}\\ \Delta u_{h,1996}^{E}=\phi^{E}\eta_{h,1996}+\psi^{E}\varepsilon_{h,1996}+\Delta \xi_{h,1996}^{E}\\ \begin{array}{c} \vdots \\ \Delta u_{h,2005}^{E}=\phi^{E}\eta_{h,2005}+\psi^{E}\varepsilon_{h,2005}+\Delta \xi_{h,2005}^{E} \end{array} \end{array}$$

We exploit the time variation in the second order moments of the income process to estimate their effects on the different expenditures, as in Blundell et al. (2008), Adda et al. (2009), Carneiro et al. (2010), among others. We assume that *η_h,t_*, *ε_h,t_*, and *ξ_h,t_* are independent and serially uncorrelated.

We use a similar framework to estimate the trivariate response model of income (Eq. (3)) and the price of calories (or energy intake) for men and women (Eqs. (5a) and (5b)). One of the main differences in these models, however, is that we model the effect of *household*-level income shocks on *individual*-level nutritional intakes, allowing for any clustering of individuals’ diets within the household. We can write out the covariance matrix for unexplained changes in energy intake for men (and similarly for women, replacing the subscript 1 with subscript 2) as:

(A.3) $$\text{cov}\left(\Delta u_{1h,t}^{C},\Delta u_{1h,t}^{C}\right)=\phi_{1}^{2}\sigma_{\eta ,t}^{2}+\psi_{1}^{2}\sigma_{\varepsilon ,t}^{2}+\sigma_{1,\xi ,t}^{2}+\sigma_{1,\xi ,t-1}^{2}\;\text{cov}\left(\Delta u_{1h,t}^{C},\Delta u_{1h,t-1}^{C}\right)=-\sigma_{1,\xi ,t-1}^{2}$$

and zero otherwise, where the superscript C again denotes energy intake (calories). Similarly, the covariance matrix for income and men’s energy intake growth is given by:

(A.4) $$\text{cov}\left(\Delta u_{h,t}^{Y},\Delta u_{1h,t}^{C}\right)=\phi_{1}\sigma_{\eta ,t}^{2}+\psi_{1}\sigma_{\varepsilon ,t}^{2}\;\text{cov}\left(\Delta u_{h,t+1}^{Y},\Delta u_{1h,t}^{C}\right)=-\psi_{1}\sigma_{\varepsilon ,t}^{2}$$

and zero otherwise. Furthermore, we can denote the covariance matrix between the two household members as:

$$\text{cov}\left(\Delta u_{1h,t}^{C},\Delta u_{2h,t}^{C}\right)=\phi_{1}\phi_{2}\sigma_{\eta ,t}^{2}+\psi_{1}\psi_{2}\sigma_{\varepsilon ,t}^{2}+\sigma_{\xi 12,t}+\sigma_{\xi 12,t-1}\;\text{cov}\left(\Delta u_{1h,t}^{C},\Delta u_{2h,t-1}^{C}\right)=\text{cov}\left(\Delta u_{1h,t-1}^{C}\Delta u_{2h,t}^{C}\right)=-\sigma_{\xi_{12},t-1}$$

and zero otherwise. Note that the above changes to a two-year difference rather than a one-year difference for 1998 and 2000; the model-implied variances and covariances adjust accordingly:

$$\text{cov}\left(\Delta u_{1h,t}^{C},\Delta u_{1h,t}^{C}\right)=\phi_{1}^{2}\left(\sigma_{\eta ,t}^{2}+\sigma_{\eta ,t-1}^{2}\right)+\psi_{1}^{2}\left(\sigma_{\varepsilon ,t}^{2}+\sigma_{\varepsilon ,t-1}^{2}\right)+\sigma_{1\xi ,t}^{2}+\sigma_{1\xi ,t-2}^{2}\;\text{cov}\left(\Delta u_{1h,t}^{C},\Delta u_{h,t}^{Y}\right)=\phi_{1}\left(\sigma_{\eta ,t}^{2}+\sigma_{\eta ,t-1}^{2}\right)+\psi_{1}\sigma_{\varepsilon ,t}^{2}$$

Despite this, *ϕ* and *ψ* remain identified. Indeed, simulations (available upon request) show that the estimates for *ϕ* and *ψ* are unbiased when the growth in unexplained income and the outcome of interest is taken over a two year period.

Note that, although measurement error is likely to affect the observed data, we do not model it here. Meghir and Pistaferri (2004) show that, although we can still identify the variance of the permanent shocks, we cannot disentangle the variance of the transitory shocks from the variance of any measurement error. Blundell et al. (2008) show that the partial insurance parameter, *ϕ*, remains identified under measurement error, while only a lower bound for *ψ* is identifiable. After some experimentation, we restrict the factor loadings to be time-invariant in all analyses. Furthermore, we assume there is no effect of dietary intake shocks on income (see e.g. Strauss and Thomas (1995) for a discussion of the nutrition effects on income). Indeed, as our sample consists of working age individuals, we do not expect any health shocks due to old age to impact on incomes.

Regarding the identification problem concerning age, period and cohort effects, we assume the latter to be zero. The large fluctuations in the Russian economy over the years examined here indeed suggest that it is important to control for period effects. Similarly, with incomes, diet and energy intakes changing over the life course, it is important to adjust for age effects. Hence, all analyses control for a full set of year dummies and a flexible (quartic polynomial) age trend of both adult household members.

In addition to the constraints specified above, we impose further constraints to identify the joint income-energy intake model. More specifically, we impose $\sigma_{i\xi ,1}^{2}=\sigma_{i\xi ,0}^{2}$, $\sigma_{i\xi ,T}^{2}=\sigma_{i\xi ,T-1}^{2}$, $\sigma_{\xi_{12},0}=\sigma_{\xi_{12},1}$ and $\sigma_{\xi_{12},T}=\sigma_{\xi_{12},T-1}$. Hence, the parameter vector to be estimated is: $\Lambda =\left(\sigma_{\varepsilon ,1}^{2},\ldots ,\sigma_{\varepsilon ,T-1}^{2},\sigma_{\eta ,1}^{2},\ldots ,\sigma_{\eta ,T}^{2},\sigma_{1\xi ,1}^{2},\ldots ,\sigma_{1\xi ,T-1}^{2},\sigma_{2\xi ,1}^{2},\ldots ,\sigma_{2\xi ,T-1}^{2},\sigma_{\xi_{12},1},\ldots \sigma_{\xi_{12},T-1},\phi_{1},\psi_{1},\phi_{2},\psi_{2}\right)$.

We formulate the model as a structural equation model and estimate the parameter vectors Λ^Y^ and Λ by full-information maximum likelihood, as implemented in the sem command within Stata. We report household-level cluster-robust standard errors.

## Appendix B Additional figures and tables

Fig. B3 compares the performance of the income-only model to the observed variances of income growth. This shows that the model-implied variances are similar to the observed income variances. They indicate a relatively small downward trend until 2000, after which they reduce substantially.

Fig. B4 presents the time-varying variances of the permanent and transitory income shocks. This shows that the variance of the permanent shocks is low and relatively stable over the observation period, whilst the variances of transitory shocks are much higher, remaining relatively constant during the economic downturn and then falling substantially. The changes in the variance of transitory shocks is consistent with the economic volatility during this period (see Section 2): factors such as wage arrears, short-time and involuntary leave were common during the downturn (represented by a higher variance in transitory shocks), but reduced substantially during the recovery. The higher variances of transitory compared to permanent shocks are also consistent with the findings by Gorodnichenko et al. (2010). Their estimates, based on the full RLMS data, range between 0.069–0.115 and 0.153–0.305 for the permanent and transitory shocks respectively. Although we use a different sample, our estimates are similar, suggesting that a one standard deviation increase in the permanent shock increases unexplained income growth by between 17 and 29% (i.e. ση ranges between 0.17–0.29).

## Footnotes

1 The nutrition literature suggests that preferences and dietary patterns are highly resistant to change (Dore et al., 2003). The amount of food that individuals eat is, in general, governed by their energy needs, where weight-stable individuals will consume enough food to satisfy their energy requirements (Scarborough et al., 2007). Hence, we argue that individuals aim to maintain a constant energy intake, as has been shown for e.g. monkeys (Hansen et al., 1981), rats (Adolph, 1947, Carlisle and Stellar, 1969), and gerbils (Kanarek et al., 1977). However, we note that there is no *universal* individual daily energy requirement, since it varies with factors such as body size, body composition, physical activity, as well as geographic, cultural and economic factors (FAO, 2001).

2 Although individuals’ dietary quality is clearly also related to their health, this paper focuses on nutritional intakes, rather than health, for two reasons. First, for many health outcomes, there is unlikely to be a contemporaneous effect of dietary intakes, as it takes time for individuals’ health to be affected by their diet, where the relevant time lag is not necessarily clear *a priori*. Second, there is a large literature that specifically explores the effects of poor diets on later life health; we refer the reader to this literature (see e.g. Kuh and Ben-Shlomo, 2007).

3 In an attempt to deal with the lack of such detailed data, Adda et al. (2009) use a synthetic cohort methodology to collect data on health and income for a 25 year period, whilst Schroeter (2008) specify a theoretical model, using income (and price) elasticities from the consumption literature to predict their effects on individual health, and Chesher (1997) estimates *individual*-level average nutrient intakes from *household*-level data.

4 The framework has also been applied in other contexts, for example examining the effects of income shocks on health and health behaviours (Adda et al., 2009), on parental investments in children (Carneiro and Ginja, 2012), and on child human capital outcomes (Carneiro et al., 2010).

5 The literature on developing countries has mixed results, with some estimating an income elasticity of the demand for energy to be close to one (e.g. Pitt, 1983, Strauss, 1984) and others close to zero (Behrman and Deolalikar, 1987, Behrman et al., 1988). For a review of this literature, see e.g. Strauss and Thomas (1995).

6 Most Russian households during our observation period did not have savings or access to financial institutions, restraining opportunities for borrowing and lending. However, as we discussed above, informal networks are very important, with the majority of households indicating to have used such a network in the last month. Hence, although the conceptual framework generally refers to *formal* exchanges, we argue that the framework can also be applied to our setting, with a larger role for the informal sector.

7 In fact, our data include a question in 1998, asking which of a list of options the respondents have done to “adjust to the new living conditions” in the past year. The most commonly reported answer is “cut down on buying clothes/shoes” (67%), followed by “cut down on meals” (55%), and “spend less money on holidays” (47%). Only 8% and 17% indicate to “find supplementary work” and “change jobs” respectively.

8 Note that expenditures are measured at the household level. Hence, if −for example- men are allocated a larger share of food spending compared to women, this will underestimate the ratio of energy to food spending for men (and overestimate it for women). Although we do allow for different parameters for men and women, this should be taken into account when interpreting the results. We also explore the effects of income shocks on the *level* of energy intake for men and women separately below.

9 The data prior to 1994 is not comparable to that from 1994 onwards, and the data on nutrition were only collected up to 2005 (see www.cpc.unc.edu/rlms for more information and data documentation).

10 Durables include household items and appliances, vehicles, garage, building materials, books, and sporting equipment. Services include transportation, tailoring, repair, laundry, postal, ritual, medical, child support, tourist, adult training courses and insurance. Utilities include payments for the apartment and utilities. Fuel includes fuel for running vehicles, firewood, coal, peat, kerosene, and bottled gas.

11 Grains include white and black (rye) bread, groat, flour, pasta; meats include canned meat, beef/veal, mutton, pork, poultry, lard, fresh and canned fish, processed meats, smoked meats, animal organs; dairy includes fresh and canned milk, butter, cheese, curd, yoghurt, margarine, eggs, cream, vegetable oil; fruit and veg include potatoes, cabbage, onions, beets, tomatoes, melons, cucumbers, squash, canned and fresh other vegetables, mushrooms, fresh, canned and dried fruit, nuts, berries, spices; sweets include ice cream, sugar, candy, jam, honey, cakes; beverages include tea, coffee, beverages, vodka, liquor, beer, tobacco.

12 Our findings are unlikely to be affected by seasonality. First, the data are collected over just a four month period. Second, Gorodnichenko et al. (2009) show that the monthly expenditures in RLMS are similar to those based on annual expenditures in the Household Budget Survey.

13 In the 1996 survey, individuals used the same method to record a 48-h (rather than 24-h) dietary recall. Due to measurement error as well as day-to-day variation in energy intake, this leads to the standard deviation of energy intake in 1996 to be 8% (men) and 13% (women) smaller than that in the other years. Our results are robust to omitting the year 1996 from the model.

14 Appendix B, Fig. B3 shows the performance of the income-only model, comparing the model-implied variances to the observed variances of income growth. Fig. B4 presents the time-varying variances of the permanent and transitory income shocks.

15 Gorodnichenko et al. (2010) find that a 10% permanent and transitory income shock leads to a 7% and 0.8% change in consumption respectively for the full sample. Although not distinguishing between permanent and transitory shocks, Stillman (2001) finds that a 10% income shock in Russia changes household’s food and total non-durable expenditures by 7–11%. Skoufias (2003) finds that food expenditures are better protected against income shocks than non-food expenditures, arguing that the latter may be an important component to smooth consumption in Russia.

16 High education is defined as having higher than secondary education. We create the ‘asset-indicator’ from information on whether the household owns a colour TV, VCR, car and fridge. Separating households by high and low income, where the former is defined as having an average income above the sample mean, gives similar results. However, as this is more sensitive to income shocks, we only present the analyses by whether the household owns assets. Being part of an informal network is defined as having given/received money/goods from friends, family members, stranger or organisations.
